# Circular RNA CHACR is involved in the pathogenesis of cardiac hypertrophy

**DOI:** 10.7150/thno.104695

**Published:** 2025-02-26

**Authors:** Lili Chen, Wenjing Wang, Yiheng Zhao, Shuchen Zhang, Xiang Zhou

**Affiliations:** 1 Central Laboratory, The Second Affiliated Hospital of Soochow University, Suzhou, China.; 2 Intensive Care Unit, The Second Affiliated Hospital of Soochow University, Suzhou, China.; 3 Department of Cardiology, The Second Affiliated Hospital of Nanjing Medical University, Nanjing, China.

**Keywords:** cardiac hypertrophy, CHACR, CPT1b, Jak2/Stat3

## Abstract

**Background:** Circular RNAs (circRNAs) exhibit differential expression in cardiac hypertrophy; however, their functions and mechanisms remain largely unexplored. This study aimed to determine the involvement of circRNAs in the pathogenesis of myocardial hypertrophy.

**Methods:** A mouse model of cardiac hypertrophy was established using transverse aortic constriction (TAC) and differentially expressed circRNAs were identified via high-throughput sequencing. To facilitate gene overexpression or knockdown, related viruses were injected into myocardial tissues of the mice. Cardiomyocyte hypertrophy was assessed using quantitative real-time PCR and immunofluorescence staining. RNA immunoprecipitation, RNA pull-down assay and fluorescence *in situ* hybridization were conducted to confirm the interaction between circRNAs and proteins. Protein expression and degradation were evaluated using cycloheximide-chase assay, immunoprecipitation, and western blotting.

**Results:** Cardiac hypertrophy-associated circRNA (CHACR) was significantly downregulated in myocardial tissues from TAC mice. CHACR can attenuate cardiac hypertrophy through upregulating carnitine palmitoyltransferase-1b (CPT1b) expression. Mechanistically, CHACR directly interacted with CPT1b and decreased its protein degradation by inhibiting the ubiquitin-proteasome pathway to increase its expression in cardiomyocytes. Moreover, CPT1b overexpression decreased L-carnitine levels and inhibited the Jak2/Stat3 signaling pathway, which was associated with the pathogenesis of myocardial hypertrophy.

**Conclusions:** CHACR attenuated cardiomyocyte hypertrophy by facilitating the expression of CPT1b, which plays a role in regulating the Jak2/Stat3 pathway via L-carnitine. CHACR may thus be a potential therapeutic target for pathological myocardial hypertrophy.

## Introduction

Myocardial hypertrophy is a compensatory response to increased cardiac afterload, which mainly manifests as cardiomyocyte enlargement, interstitial fibrosis, increased collagen synthesis, and activation of myofibroblasts, eventually resulting in ventricular remodeling and heart failure 1,2. Pathological cardiac hypertrophy contributes independently to the risk of higher cardiovascular morbidity and mortality 3,4. However, the molecular mechanisms and therapeutic targets of myocardial hypertrophy are still poorly understood.

Circular RNAs (circRNAs) are RNA molecules with a unique covalently closed loop structure, granting them significant stability and protection against exonuclease degradation. Previous research demonstrated that circRNAs can function as microRNA (miRNA) sponges to participate in the pathogenesis of the cardiovascular diseases 5-7. Specifically, heart-related circRNA is a competing endogenous RNA that inhibited myocardial hypertrophy through binding to miR-223 and subsequently promoting the expression of apoptosis repressor with a CARD domain 8. In addition to serving as miRNA sponges, accumulating evidence indicated that circRNAs can also interact with transcription factors or proteins, thereby exerting transcriptional regulatory functions 9-10. For instance, circYap has been shown to bind directly to tropomyosin-4 and gamma-actin, facilitating their interaction, which suppresses actin polymerization and subsequent fibrosis, ultimately exerting a negative effect on cardiac hypertrophy 11. Understanding the involvement of circRNAs in myocardial hypertrophy has developed as a key topic of interest in recent years.

In this study, we identified a new cardiac hypertrophy-associated circRNA (CHACR) using high-throughput sequencing. Our findings indicate that CHACR mitigated cardiac hypertrophy by regulating the expression of carnitine palmitoyltransferase-1b (CPT1b) protein. We further investigated its molecular mechanisms underlying myocardial hypertrophy, which provided a deeper understanding of circRNA-protein interaction and highlighted the promise of CHACR as a therapeutic target for pathological cardiac hypertrophy.

## Materials and methods

### Transverse aortic constriction (TAC)

The study involved male C57BL/6 mice, aged between 8 and 10 weeks, which were procured from the Shanghai Laboratory Animal Center in China and randomly assigned to either the control or TAC group. The mice were anesthetized with a cocktail of ketamine (100 mg/kg) and xylazine (2.5 mg/kg). The aortic arch was visualized through a left anterior thoracotomy. A TAC model was created by placing a 6-0 stitch near the aorta and inserting a 27-gauge needle between the left and right carotid arteries. The needle was swiftly removed, and 4-0 stitch was used to suture the wound. The control group mice underwent the same procedure without tying the suture. The mice were kept on a temperature-controlled blanket post-surgery until they were fully recovered. Cervical dislocation was used to euthanize the mice for the following experiments. All experiments were approved by the Animal Ethics Committee of Soochow University.

### Virus injection

Adeno-associated virus serotype 9 (AAV9) vectors were used to achieve CHACR overexpression, and adenovirus vector (ADV) was applied to interfere with the expression of CPT1b in the mouse models. AAV9-CHACR and ADV-shCPT1b-1# (labeled ADV-shCPT1b), together with their control vectors (AAV9-NC and ADV-shNC, respectively), were purchased from GenePharma (Shanghai, China).

The mice were anesthetized as described above. During TAC surgery, 8 mice per group received myocardial injections of either AAV9-CHACR or AAV9-NC at a dose of 5×10^10^ vg per mouse. Seven days post-TAC surgery, myocardial injections of ADV-shCPT1b or ADV-shNC (5×10^8^ vg per mouse) were administered. The virus was rapidly injected at 3-5 sites in the thicker myocardium using a microinjector. Subsequently, the operator carefully repositioned the heart in the thoracic cavity, expelled the air by squeezing it, and secured the ligation incision. The collected heart samples were used for histological and functional analyses after echocardiography examination. After receiving an intraperitoneal injection of pentobarbital (200 mg/kg), the mice were euthanized via cervical dislocation, and their hearts were swiftly extracted for weighing and further study.

To overexpress CHACR or CPT1b alone, AAV9-CHACR, AAV9-CPT1b, and AAV9-NC were administered intravenously to mice 3 days following TAC. In the same way, the hearts were dissected and employed for other assessments as mentioned above.

### Echocardiography

Four weeks post-TAC, a double-blind mouse echocardiography was conducted using the Vevo 2100 imaging system (Fujifilm VisualSonics, Toronto, Canada). Mice were anesthetized using 2% isoflurane. The probe was positioned on the left sternum to visualize the horizontal mitral valve in the long-axis view of the left ventricle. Left ventricular posterior end-diastolic wall thickness (LVPWd), left ventricular posterior end-systolic wall thickness (LVPWs), left ventricular ejection fraction (LVEF), and left ventricular fractional shortening (LVFS) were subsequently measured, respectively. An experienced technician averaged all measurements over three consecutive cardiac cycles.

### The circRNA-seq

The extraction of total RNA samples from mouse hearts was completed by using TRIzol reagent (Invitrogen, Carlsbad, CA, USA). Total RNA was quantified following processing and 5 μg of total RNA was utilized for circRNA extraction. Subsequently, these RNAs were constructed into a circRNA-seq library. Library quality and quantity were assessed and chemically fragmented into single-stranded RNA. Using Illumina flow cells, the libraries were clustered *in situ* and sequenced for 150 cycles with an Illumina HiSeq sequencer. Sequencing was conducted by Cloudseq Inc., and circRNA-seq reads were qualitatively visualized using the Illumina HiSeq 4000 sequencer. Reads were processed by trimming the 3ʹ-adaptor and filtering out low-quality sequences using Cutadapt (v1.9.3) 12, followed by alignment to the reference genome/transcriptome with STAR 13. CircRNAs were detected and annotated with DCC software 14, and validated circRNAs were further annotated based on the circBase 15 and circ2Trait 16 disease databases. The raw junction reads for all samples were normalized in line with the number of total reads and then log2 transformed. The t-test was executed to identify the differentially expressed circRNAs. CircRNAs with differentially expression were identified using a p-value threshold of ≤ 0.05 and an absolute fold change of ≥ 2. Selected target circRNAs underwent qPCR validation and structural verification to assess their expression patterns.

### Cardiomyocyte culture and measurement of cell surface area

Cardiomyocytes were isolated from one-day-old mice (Shanghai Laboratory Animal Center). Following thoracotomies under pentobarbital anesthesia, the hearts were excised and isolated from adipose tissue and blood vessels using Dulbecco's Modified Eagle's Medium (DMEM, Gibco, Grand Island, NY, USA). Tissue fragments were collected by shearing the hearts and digested in 0.125% trypsin at 37 °C. Then, 0.05% collagenase (Sigma-Aldrich, St. Louis, MO, USA) was used to redigested the separated cell suspensions. The digestion was halted using DMEM with 10% fetal bovine serum (FBS, Gibco). The cell pellets were acquired by centrifuging the above cell suspensions at 2,000 rpm for 10 min after discarding the supernatant. Cells were obtained and resuspended, followed by a 1-hour pre-plating to eliminate fibroblasts. Non-adherent cardiomyocytes were seeded at a concentration of 1×10^6^ cells per well onto a culture plate with 1% 5-bromodeoxyuridine. Cardiomyocytes were processed after 48 h of culture, where hypertrophy was established by incubating them with Ang II (1 μg/mL) for 24 h.

The measurement of cell surface area was conducted by capturing images on a confocal laser microscope (Zeiss, Oberkochen, Germany) after staining with α-actinin (Abcam, Cambridge, UK). Briefly, cardiomyocytes were immobilized using 4% formaldehyde, following treatment with 0.1%Triton X-100 in PBS for permeabilization. Cells were incubated overnight with anti-α-actinin, then treated with anti-RFP antibodies (Abcam), and finally incubated with DAPI for 10 minutes at room temperature. The relative cell size was analyzed using Image-Pro Plus software by randomly selecting 50-100 cardiomyocytes from each group.

### RNA extraction and quantitative real-time PCR (qRT-PCR)

RNA was extracted from hypertrophic cardiomyocytes by the Cytoplasmic & Nuclear RNA Purification Kit (SKU37400, Norgen Biotek, Canada) to isolate nuclear and cytoplasmic RNA. The concentration was measured to calculate the total RNA in nucleus and cytoplasm with qRT-PCR analysis. Cardiomyocytes or hearts were collected for total RNA extraction as outlined below. Specially, RNA samples after DNAse I treatment (Takara, Japan) were quantified and reverse transcribed using HiScript III RT SuperMix for qPCR with gDNA wiper (Vazyme, Nanjing, China). Amplification was completed on the Applied Biosystems QuantStudio 3 (Thermo Fisher Scientific, Waltham, USA) using ChamQ Universal SYBR qPCR Master Mix (Vazyme). The sequences of circRNA were obtained from NCBI, and divergent PCR primers were designed around the junction sites to ensure coverage by the PCR product, with primers evenly distributed on either side. The relative expression level was normalized to the reference genes using a relative standard curve. The primer sequences of genes are listed in **Table S1**.

### RNase R

RNase R was used to verify the properties of circRNA. RNAs were extracted from cardiomyocytes and split into two equal parts for RNase R and Mock samples. Briefly, RNA (2 mg) samples were incubated with RNase R (3 U/μg, RNR07250, Epicentre, Madison, WI, USA) for 30 min at 37 °C, and qRT-PCR was carried out to determine the RNA expression levels of CHACR.

### Transduction and RNA interference

For CHACR or CPT1b overexpression, cardiomyocytes were incubated for 24 h with lentiviruses (1 × 10^8^ viral particles in 1% serum medium (GenePharma)). The next step was performed after incubation with 1% serum medium for another 24 h. Then, siRNA oligonucleotides specific for CPT1b were synthesized by GenePharma. The following siRNA sequences were used: CPT1b-1# siRNA, sense-5ʹ-GGUAUGGUGCCAUGUGCUTT-3ʹ, antisense-5ʹ-AGCACAUGGGCACCAUACCTT-3ʹ; CPT1b-2# siRNA, sense-5ʹ-GCAUCCCAGGCAAAGAGACTT-3ʹ, antisense-5ʹ-GUCUCUUUGCCUGGGAUGCTT-3ʹ; and NC siRNA, sense-5ʹ-UUCUCCGAACGUGUCACGUTT-3ʹ, antisense-5ʹ-ACGUGACACGUUCGGAGAATT-3ʹ. The siRNA transfections were completed using Lipofectamine 3000 (Invitrogen).

### Western blotting

Total protein samples were obtained using RIPA lysis (Beyotime, Shanghai, China) with protease inhibitors (CAT-4906845001, Roche, Basel, Switzerland). Proteins in the nucleus or cytoplasm were isolated in accordance with the manufacturer's instructions (Thermo Fisher Scientific). The BCA assay was applied to determine the protein concentrations. SDS-PAGE was used to separate proteins with discrepant molecular weight. These proteins were then transferred to polyvinylidene fluoride membranes, then incubated with 5% bovine serum albumin in 1× Tris-buffered saline (TBS) and 0.1% Tween 20 (TBS-T) at room temperature with continuous shaking for 2 h. Next, the membranes were incubated with primary antibodies, including GAPDH (1:1000, 60004-1-Ig, Proteintech, Chicago, IL, USA), CPT1b (1:1000, ab134135, Abcam), phospho-Signal Transducer and Activator of Transcription 3 (Stat3) (Tyr705) (1:1000, #9145, CST, Beverly, MA, USA), Stat3 (1:1000, #30385, CST), phospho-Jak2 (Tyr1007/1008) (1:1000, #3771, CST), and Jak2 (1:1000, #3230, CST) overnight at 4 °C. Then, the membranes were washed three times with TBS-T, after which the appropriate secondary antibodies (Proteintech) were used to incubate the membranes at room temperature for 1.5 h, following washing three times with TBS-T again. Immune complex detection on the blots was performed on an enhanced chemiluminescence system (GelDoc XR+, Bio-Rad, USA). Image J software was used for quantitative analysis.

### Cycloheximide (CHX)-chase assay

CHX (S7418, Selleck Chemicals, Houston, USA) is an inhibitor of protein synthesis that was used in CHX-chase assay. CHX (20 µg/mL) treatment was conducted in cells after infecting with CHACR or NC virus for 48 h. Then, western blot analysis was applied to detect CPT1b protein expression levels at 0, 0.25, 0.5, 1, and 2 h.

### Ubiquitination assay

The transfection of cardiomyocytes with a hemagglutinin-labeled ubiquitin (HA-Ub) vector, with or without the CHACR virus, was conducted for 48 h, after which they were treated with MG132 (Selleck Chemicals, 20 μM) for 2 h. Next, the cells were lysed in RIPA lysis buffer (Beyotime) and the lysates were combined with CPT1b antibody and Protein A/G beads (Life Technologies, Carlsbad, USA) with the purpose of immunoprecipitation. Western blotting was used to determine the proteins expression level.

### RNA immunoprecipitation

To confirm RNA-protein interactions, RNA immunoprecipitation (RIP) analysis was carried out using the RIP kit (17-701, Millipore, Boston, USA). Briefly, the cell pellets were resuspended in RIP lysis buffer with protease and RNase inhibitors. Cardiomyocyte lysates were then incubated with CPT1b and IgG (Proteintech) control antibodies. Then, the RNA-protein complexes were eluted using the proteinase K buffer for purification and evaluated by qRT-PCR.

### RNA pull-down assay

RNA pull-down assays were performed by a Pierce Magnetic RNA-Protein pull-down kit (20164, ThermoFisher Scientific). Probes, including* in vitro* transcribed CHACR (5'-Biotin-aaaTAGCTTGCACCTGAGTAGCTTATTATGTACAAAAG-3') or negative control (NC) (5'-Biotin-aaaCTTTTGTACATAATAAGCTACTCAGGTGCAAGCTA-3'), were developed for pull-down assays. Briefly, 10^7^ cells were lysed in immunoprecipitation lysis buffer (1 mL) and the supernatant was separated by centrifuging at 4 °C. Probe-coated beads were constructed using 5 μg of Biotinylated CHACR or NC, mixed with cell lysis supernatant, and washed five times with a washing buffer. For more detailed analysis, bound proteins and RNAs were purified from the materials obtained through pull-down. Purified CHACR pull-down materials (labelled CHACR^+^) and the NC (labelled CHACR^-^) were sent to a commercial laboratory (Cloudseq Inc.) for LC-MS/MS analysis. The Gene Ontology (GO) enrichment analysis of the differentially expressed proteins' host genes was conducted using clusterProfiler in R package. The top 30 GO terms and pathways based on the p-values of ≤ 0.05 were selected. KEGG pathway analysis on the bound protein in the RNA pull-down materials was conducted.

### Histological analysis

For histological examination, 5-μm sections of mouse hearts were stained using hematoxylin and eosin (H&E). To assess cell cross-sectional areas, FITC-labeled wheat germ agglutinin (WGA, L4895, Sigma-Aldrich) staining was employed. The heart sections were stained using standard Masson's trichrome (HT15, Sigma-Aldrich) to assess cardiac fibrosis.

The procedure for immunofluorescence staining of Stat3 in cardiomyocytes followed the same treatment as α-actinin staining. Specifically, cells were exposed to anti-Stat3 antibodies (CST) overnight at 4 °C, following staining with anti-rabbit GFP antibodies (Abcam). Images were acquired using LSM 800 confocal laser microscope.

### IF-fluorescence *in situ* hybridization (IF-FISH)

The Fluorescence *in Situ* Hybridization Kit for RNA (R0306S, Beyotime) was applied to label protein-RNA interaction. Specifically, cardiomyocytes were treated with hybridization solution containing the CHACR-probe (5'-Cy3-GCTTGCACCTGAGTAGCTTATTATGTACAAA-3') at 60°C for 5 h and subsequently stained with CPT1b and TOM20 (a mitochondrial membrane marker). The images were obtained using a confocal laser microscope (LSM 800, Zeiss).

### Fatty acid oxidation (FAO) assay

The FAO assay kit (#BR00001; Assay Genie, Dublin, Ireland) was used to measure FAO activity. In detail, cardiomyocytes were collected and lysed in 1x cell lysis solution for 5 min, then the lysate was centrifuged at 14,000 rpm in a cold microfuge for 5 min and the supernatant was harvested. The BCA protein assay method was used to detect lysate protein concentration. Next, we performed enzyme assay by adding 20 μL of each sample to a 96-well plate (uncoated) on ice in duplicate, following adding 50 µL control solution to one set of wells and 50 µL reaction solution to the other set of wells. Then the plate was incubated in 37 °C for 60 min and cherry red color was appeared in wells. Finally, the plate was detected at 492 nm on the microplate reader (Cytation 5, Bio Tek, Vermont, USA). FAO activity was calculated and presented as units/µg proteins according to the manufacturer's instructions.

### ATP measurement

For ATP measurement in cardiomyocytes, ATP assay kit (S0026, Beyotime) was used. Briefly, cells were lysed in lysis buffer and centrifuged at 4 °C, 12,000 × g for 5 min to acquire the supernatant. Then 100 µL detection solution was added in the 96-well plate, following adding 20 µL sample supernatant or ATP standard. The reader was acquired by measuring RLU value on luminometer (Cytation 5, Bio Tek). The lysate protein concentration was determined by BCA assay and ATP production was presented as nmol/mg proteins.

### Quantification of L-carnitine

L-carnitine in cardiomyocytes was quantified using the L-Carnitine Assay Kit (MAK063, Sigma-Aldrich). Briefly, cells (1×10^6^) were homogenized and centrifuged to remove the precipitation. 50 μL of sample and 50 μL of the appropriate reaction mix was successively added to each of the wells. Then the plate was incubated for 30 min after mixing well by pipetting. The fluorescence intensity was determined at λex = 535/λem = 587 nm on fluorescence microplate reader (Cytation 5, Bio Tek).

### Statistical analysis

The data are represented as the mean ± SEM for each group. Statistical significance between two groups was determined using the Student's *t*-test with unpaired two-sided tests, and one-way or two-way ANOVA was used for comparison of three or more groups using GraphPad Prism 9. p-values of < 0.05 were considered statistically significant.

## Results

### The CHACR expression was downregulated during TAC-induced myocardial hypertrophy

**Figure 1:**
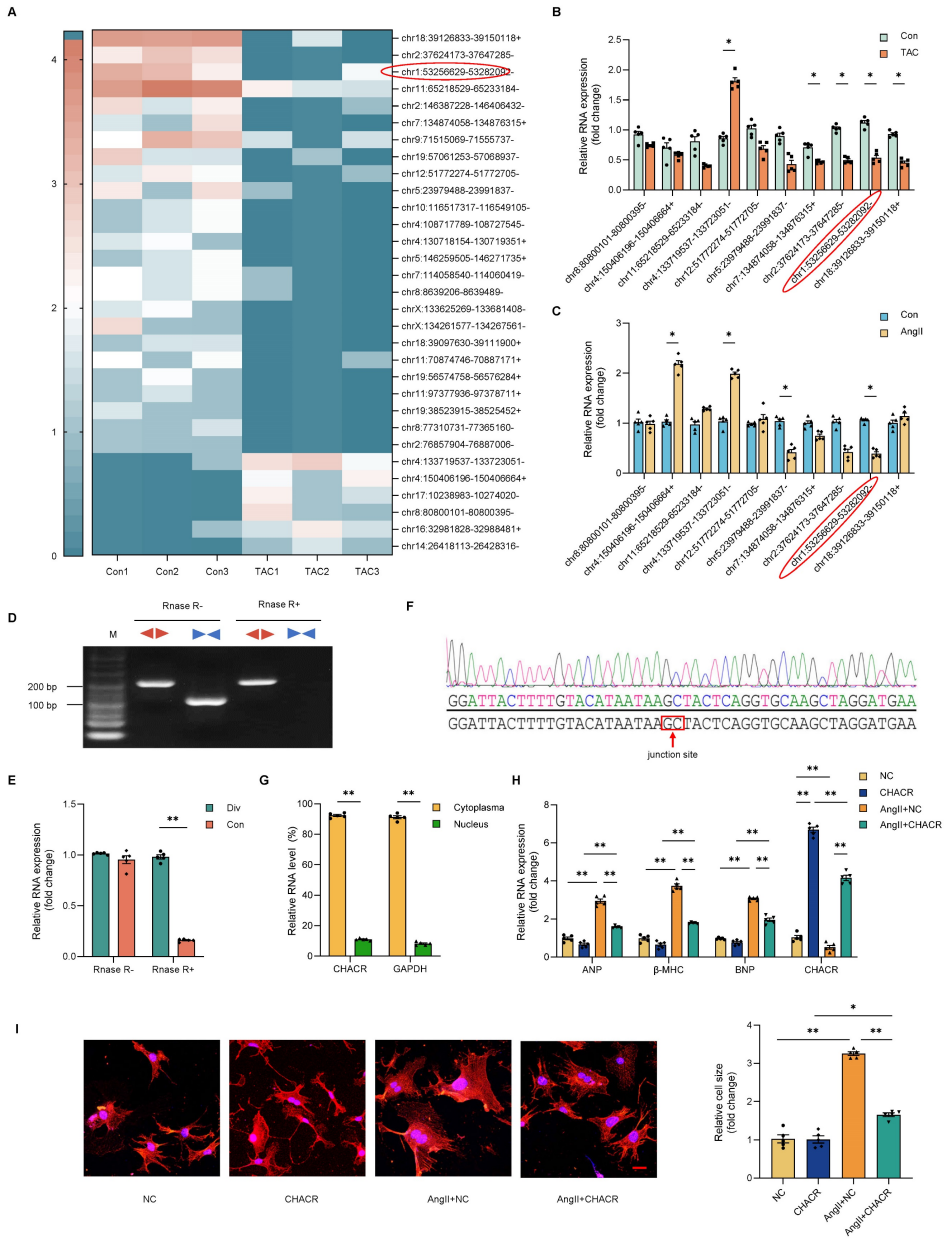
** The CHACR overexpression ameliorates cardiomyocyte hypertrophy.** (**A**) Hierarchical cluster analysis of circRNAs differentially expressed in the transverse aortic constriction (TAC) and control groups (n = 3). (**B, C**) The expression levels in cardiac tissue of TAC and control mice (n = 5), and in cardiomyocytes treated with or without Angiotensin II (Ang II,1 μg/mL) (n = 5). (**D**) PCR was performed to identify circRNA or linear RNA in cardiomyocytes treated with RNase R (n = 5). Convergent (Con, blue) and divergent (Div, red) primers were utilized to amplify linear or back-splicing products. (**E**) Quantitative real-time PCR (qRT-PCR) analysis of CHACR expression in cardiomyocytes treated with RNase R (n = 5). (**F**) DNA sequencing to identify the junction site of CHACR. (**G**) qRT-PCR analysis of CHACR expression in the cytoplasm and nuclear fraction of cardiomyocytes (n = 5). (**H**) Expression levels of hypertrophy markers in different groups of cardiomyocytes (n = 5). (**I**) Left: Effect of CHACR overexpression on cell cross-sectional area assessed by measuring sarcomere organization; scale bar, 20 μm. Right: Cell surface area quantification (n = 8). *p < 0.05, **p < 0.01.

Differences in circRNAs during TAC-induced cardiac hypertrophy were assessed using circRNA-seq assays in mouse hearts. Among 4,510 circRNAs detected, 31 circRNAs showed differential expression. Of these 31 circRNAs, 25 circRNAs were downregulated and six were upregulated (**Fig. 1A**). Ten circRNAs with significantly differential expression were selected for qRT-PCR analysis for further validation. Two circRNAs (chr1: 53256629-53282092- and chr4:133719537-133723051-) were conclusively found to be differentially expressed in the hearts of mice (**Fig. 1B**) and cardiomyocytes (**Fig. 1C**). Selected for further analysis was the circRNA chr1: 53256629-53282092-, named cardiac hypertrophy-associated circRNA (CHACR), which is analogous to hsa_circ_0001083 in humans. The sequence of CHACR, positioned on chromosome 1 of the mouse genome Pms1, is listed in **Table S2**. For the verification of CHACR as a circular RNA, convergent and divergent primers were designed to specifically amplify the canonical or back-spliced forms of Pms1. RT-PCR using divergent primers (red) demonstrated that CHACR resists digestion by RNase-R, an exoribonuclease that that breaks down linear RNAs, whereas the PCR products from linear Pms1 mRNA amplified with convergent primers (blue) were eliminated after RNase-R treatment (**Fig. 1D, E**). The circular structure of CHACR was confirmed by Sanger sequencing (**Fig. 1F**).

### Overexpression of CHACR ameliorates cardiomyocyte hypertrophy

We investigated whether CHACR regulated cardiomyocyte hypertrophy induced by Ang II. CHACR expression was predominantly localized in the cytoplasm of cardiomyocytes, rather than nucleus (**Fig. 1G**). Also, the mRNA expression levels of cardiac hypertrophy markers such as atrial natriuretic factor (ANP), myosin heavy chain-β (β-MHC), and B-type natriuretic peptide (BNP) were elevated in cardiomyocytes after Ang II stimulation (**Fig. 1H**). On the other hand, CHACR overexpression prevented Ang II-induced hypertrophy, as revealed by the decreased size of the cardiomyocyte surface areas (**Fig. 1I**). Furthermore, the mRNA expression levels of ANP, β-MHC, and BNP decreased following CHACR overexpression in hypertrophic cardiomyocytes (**Fig. 1H**). The findings suggested that overexpressing CHACR hindered the hypertrophy of cardiomyocytes caused by Ang II.

### The CHACR promotes CPT1b expression by inhibiting proteasome degradation to regulate cardiomyocyte hypertrophy

**Figure 2:**
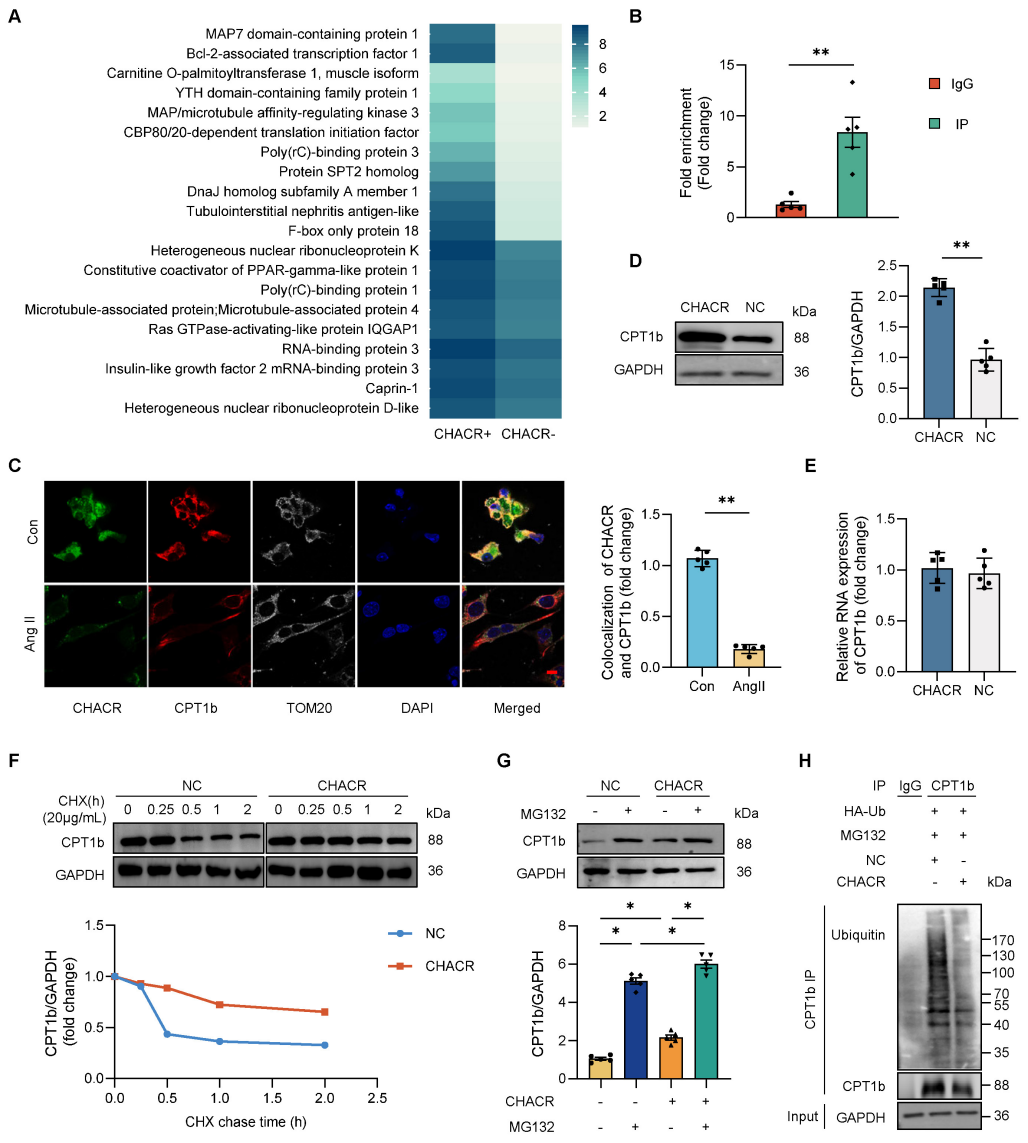
** The CHACR facilitates CPT1b protein expression by protecting CPT1b from proteasomal degradation.** (**A**) Hierarchical cluster analysis of 30 proteins differentially expressed in CHACR-positive (CHACR^+^) and CHACR-negative (CHACR^-^) groups differentiated by CHACR RNA pull-down assay. (**B**) RNA immunoprecipitation (RIP) analysis showing direct binding of CHACR to CPT1b (n = 5). (**C**) Immunofluorescence -fluorescence *in situ* hybridization (IF-FISH) results showing co-localization of CHACR and CPT1b (n = 5). Scale bar, 10 μm. (**D**) Western blot results for CPT1b expression in cardiomyocytes with or without CHACR overexpression (n = 5). (**E**) qRT-PCR analysis of CPT1b in cardiomyocytes with or without CHACR overexpression (n = 5). Western blot results for CPT1b protein levels in cardiomyocytes with or without CHACR overexpression measured after treatment with (**F**) cycloheximide (CHX; 20 μg/mL) at different time points and (**G**) MG-132 (20 μM) for 2 h (n = 5). (**H**) Cardiomyocytes transfected with ubiquitin (Ub) and CHACR after treatment with MG-132 were used to perform immunoprecipitation assay with CPT1b antibody by incubation with ubiquitin antibody (n = 5). CPT1b: carnitine palmitoyltransferase-1b. *p < 0.05, **p < 0.01.

The molecular mechanisms by which CHACR regulates cardiomyocyte hypertrophy were assessed. An RNA-protein pull-down assay in cardiomyocytes was performed with biotinylated CHACR. Some proteins, excluding AGO-2, which is an indicator protein that acts as a sponge for circRNAs, were specifically enriched in the CHACR^+^ group (**Fig. 2A**). We selected top three proteins, Map7 domain-containing protein 1 (Map7D1) 17, Bcl-2-associated transcription factor 1 (Bclaf1) 18,19 and CPT1b 20,21, which were closely related to cardiovascular diseases, for further investigation. Expression levels of CPT1b were lower in hypertrophic cardiomyocytes stimulated with Ang II compared with normal cells, excluding Map7D1 or Bclaf1. GO functional analysis revealed that CPT1b participated in most cellular processes, including metabolic processes and organic substance metabolism (**Fig. S1A**). To validate the binding of CPT1b and CHACR, immunoprecipitated RNA-binding CPT1b in cells was analyzed using the RIP assay (**Fig. 2B**). The results showed that the amount of CHACR precipitated by CPT1b was significantly greater than that precipitated by IgG, suggesting that CPT1b bound directly to CHACR. IF-FISH results also showed that CPT1b and CHACR were co-located in cardiomyocytes, and their binding was decreased when the cells were treated with Ang II mainly due to the low expression (**Fig. 2C**).

Western blot assays showed that overexpressed CHACR elevated CPT1b protein expression (**Fig. 2D**), but not mRNA expression (**Fig. 2E**). Ang II treatment also downregulated the CPT1b expression in cardiomyocytes (**Fig. S1B**). These results prompted that the protein degradation might be involved in the process of CHACR-facilitated CPT1b protein expression. The CHX assay results demonstrated that the half-life of CPT1b protein in the CHACR-overexpressed group was longer than that in the control group (**Fig. 2F**), suggesting that CHACR reduced CPT1b protein degradation in cardiomyocytes. Moreover, the MG-132 assay was used to explore whether CHACR affected CPT1b protein stability. Pre-treatment with MG-132, a proteasome inhibitor, blocked CPT1b protein degradation while CHACR overexpression further inhibited the degradation process in cardiomyocytes (**Fig. 2G**). Moreover, to investigate whether CHACR blocks the degradation of CPT1b protein through a ubiquitination-dependent mechanism, a co-immunoprecipitation assay was carried out in cardiomyocytes co-transfected with CHACR and ubiquitin. The results showed that CHACR overexpression significantly decreased the levels of ubiquitinated CPT1b protein, which protected CPT1b from proteasomal degradation (**Fig. 2H**). Thus, CHACR prevented CPT1b proteasomal degradation by interacting directly with the protein, thereby regulating its expression.

**Figure 3:**
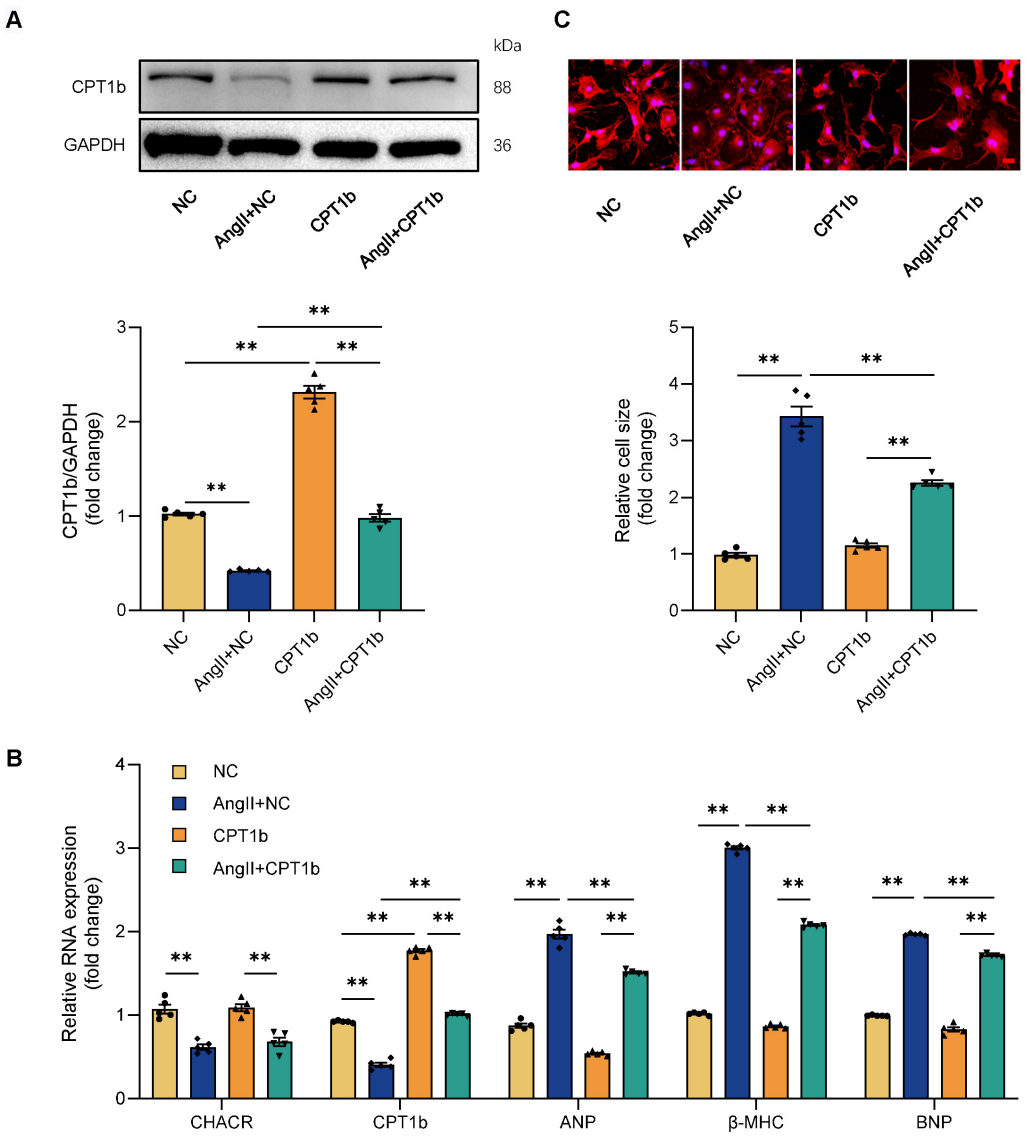
** CPT1b overexpression prevents cardiomyocyte hypertrophy induced by Ang II.** (**A**) Western blot results for CPT1b expression in cardiomyocytes with or without CPT1b overexpression and Ang II treatment (1 μg/mL) (n = 5). (**B**) qRT-PCR analysis of CHACR, CPT1b, atrial natriuretic factor (ANP), myosin heavy chain-β (β-MHC), and B-type natriuretic peptide (BNP) expression in cardiomyocytes of different groups (n = 5). (**C**) Upper: Cardiomyocyte hypertrophy assessed by measuring sarcomere organization. Scale bar, 20 μm. Lower: Cell surface area quantification (n = 5). **p < 0.01.

**Figure 4:**
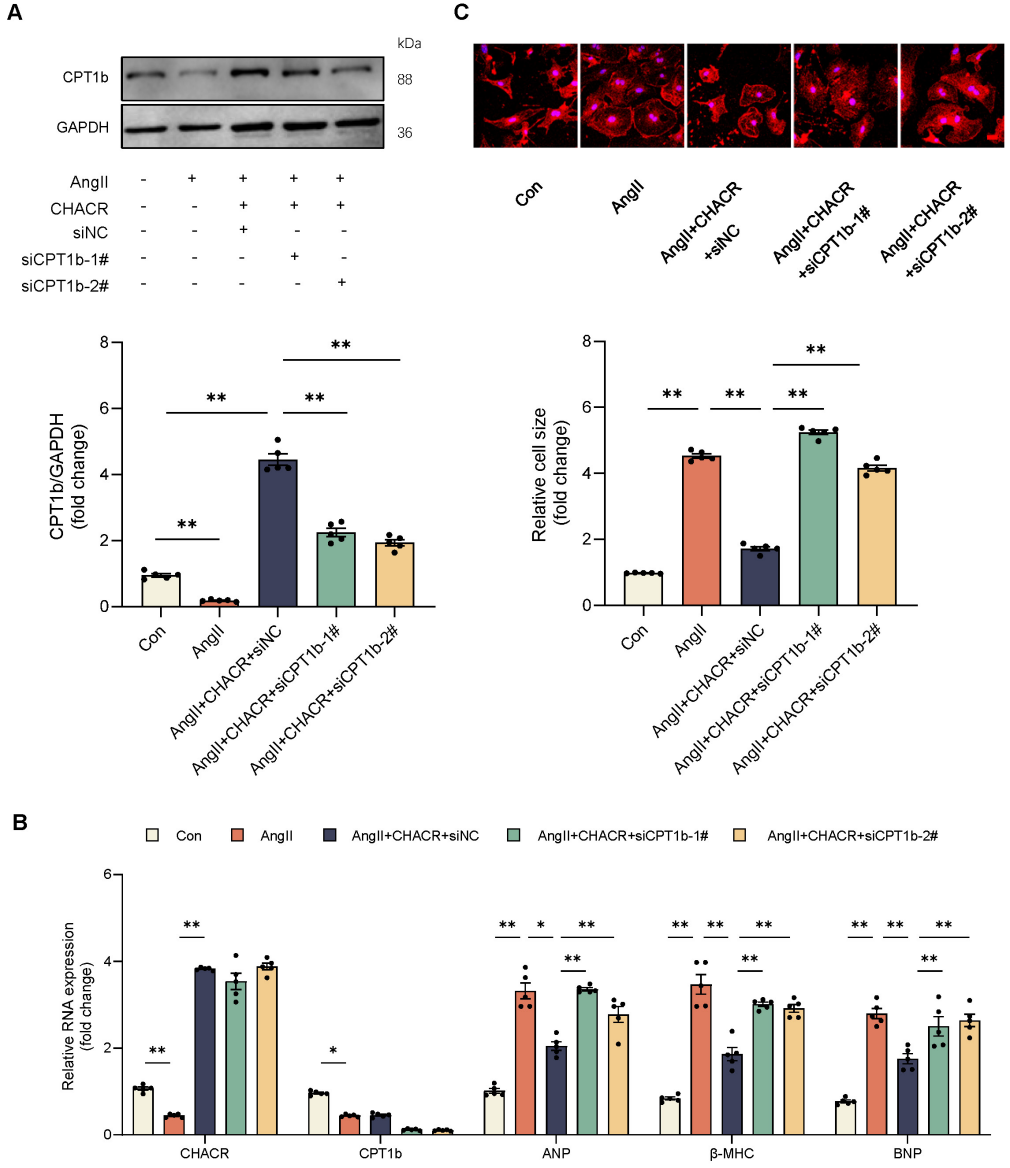
** The CHACR regulates cardiomyocyte hypertrophy through CPT1b.** (**A**) Western blot results for CPT1b expression in cardiomyocytes stimulated with Ang II (1 μg/mL) and transfected with CHACR and/or siCPT1b (n = 5). (**B**) qRT-PCR analysis of CHACR, CPT1b, ANP, β-MHC, and BNP expression in cardiomyocytes of different groups (n = 5). (**C**) Upper: Hypertrophy assessed by measuring sarcomere organization. Scale bar, 20 μm. Lower: Cell surface area quantification (n = 5). *p < 0.05, **p < 0.01.

It was then hypothesized that CHACR regulated cardiac hypertrophy through CPT1b. CPT1b protein expression was downregulated in cardiomyocytes treated by Ang II and was increased again in CPT1b virus-treated cells (**Fig. 3A**). Ang II treatment led to elevated levels of hypertrophy markers such as ANP, β-MHC, and BNP, along with increased cell surface areas in cardiomyocytes, whereas CPT1b overexpression lowered these hypertrophy markers and reduced cell surface areas (**Fig. 3B, C**). The results indicated that CPT1b overexpression ameliorated Ang II-induced cardiomyocyte hypertrophy. Moreover, CHACR overexpression in hypertrophic cells increased CPT1b protein expression and alleviated cardiomyocyte hypertrophy, which was aggravated by CPT1b knockdown. CHACR thus suppressed Ang II-induced cardiomyocyte hypertrophy by regulating CPT1b expression (**Fig. 4A-C**).

### The CHACR regulates cardiomyocyte hypertrophy through the Jak2/Stat3 signaling pathway

We conducted KEGG pathway analysis on the bound protein in the RNA pull-down materials, which showed that fatty acid metabolism and JAK pathway was involved (**Fig. S2A**). It was found that CPT1b knockdown down-regulated fatty acid oxidation activity (**Fig. S2B**) and ATP production (**Fig. S2C**), which was extensively researched in recent years 20,22. Therefore, we focused on Jak2/Stat3 signaling pathway, which was a vital signaling pathway involved in cardiac hypertrophy.

**Figure 5:**
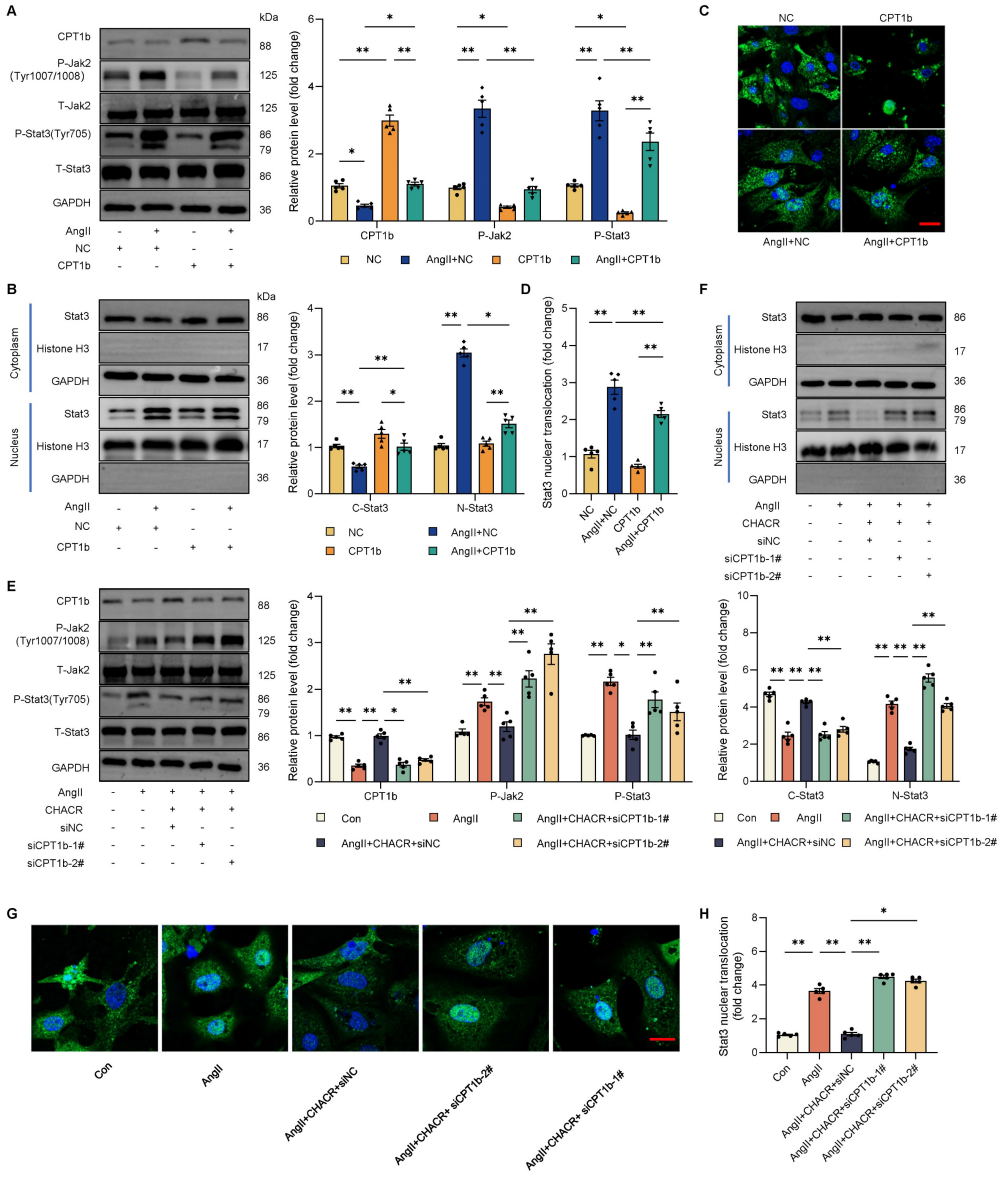
** The CHACR inhibits the Jak2/Stat3 pathway by regulating CPT1b expression in cardiomyocytes.** (**A**) Western blot results showing CPT1b, P-Jak2 (Tyr1007/1008), and P-Stat3 (Tyr705) expression in cardiomyocytes transfected with CPT1b (n = 5). (**B**) Protein expressions of Stat3 in nucleus or cytoplasm were detected by western blot assay (n = 5). (**C**) Visualization of Stat3 subcellular localization using confocal microscopy. (**D**) Quantitative analysis of relative Stat3 nuclear translocation. Scale bar, 20 μm (n = 5). (**E**) Western blot results showing CPT1b, P-Jak2 (Tyr1007/1008), and P-Stat3 (Tyr705) expression in cardiomyocytes transfected with CHACR or siCPT1b (n = 5). (**F**) Protein expressions of Stat3 in nucleus or cytoplasm were determined by western blot assay (n = 5). (**G**) Visualization of Stat3 subcellular localization was conducted using immunofluorescence assay. (**H**) Quantitative analysis of relative Stat3 nuclear translocation. Scale bar, 20 μm (n = 5). Jak2: Janus kinase 2; Stat3: Signal Transducer and Activator of Transcription 3. *p < 0.05, **p < 0.01.

To further investigated whether CPT1b regulated cardiomyocyte hypertrophy *via* the Jak2/Stat3 signaling pathway, western blot assay was performed in cardiomyocytes. Results showed that Ang II increased the phosphorylation levels of Jak2 (Tyr1007/1008) and Stat3 (Tyr705), while CPT1b protein expression was downregulated. In contrast, the protein expression levels of Jak2 and Stat3 remained unchanged. However, the phosphorylation levels of Jak2 and Stat3 decreased in CPT1b-overexpressing hypertrophic cardiomyocytes (**Fig. 5A**). Western blot assay and IF staining results showed that Ang II promoted nuclear import of Stat3, which was reversed after CPT1b overexpression in cardiomyocytes (**Fig. 5B-D**).

Given that CHACR regulated cardiomyocyte hypertrophy by controlling CPT1b expression, the role of CHACR in influencing the Jak2/Stat3 signaling pathway was investigated further. As expected, CHACR overexpression in hypertrophic cardiomyocytes restrained the phosphorylation levels of Jak2 and Stat3, while the phosphorylation levels were re-enhanced after CPT1b knockdown by siRNA transfection (**Fig. 5E**). Similarly, CHACR suppressed the nuclear import of Stat3, which was increased in hypertrophic cardiomyocytes transfected with siCPT1b (**Fig. 5F-H**). These observations suggested that Ang II-induced downregulation of CHACR activated the Jak2/Stat3 signaling pathway to aggravate hypertrophy by suppressing CPT1b expression.

**Figure 6:**
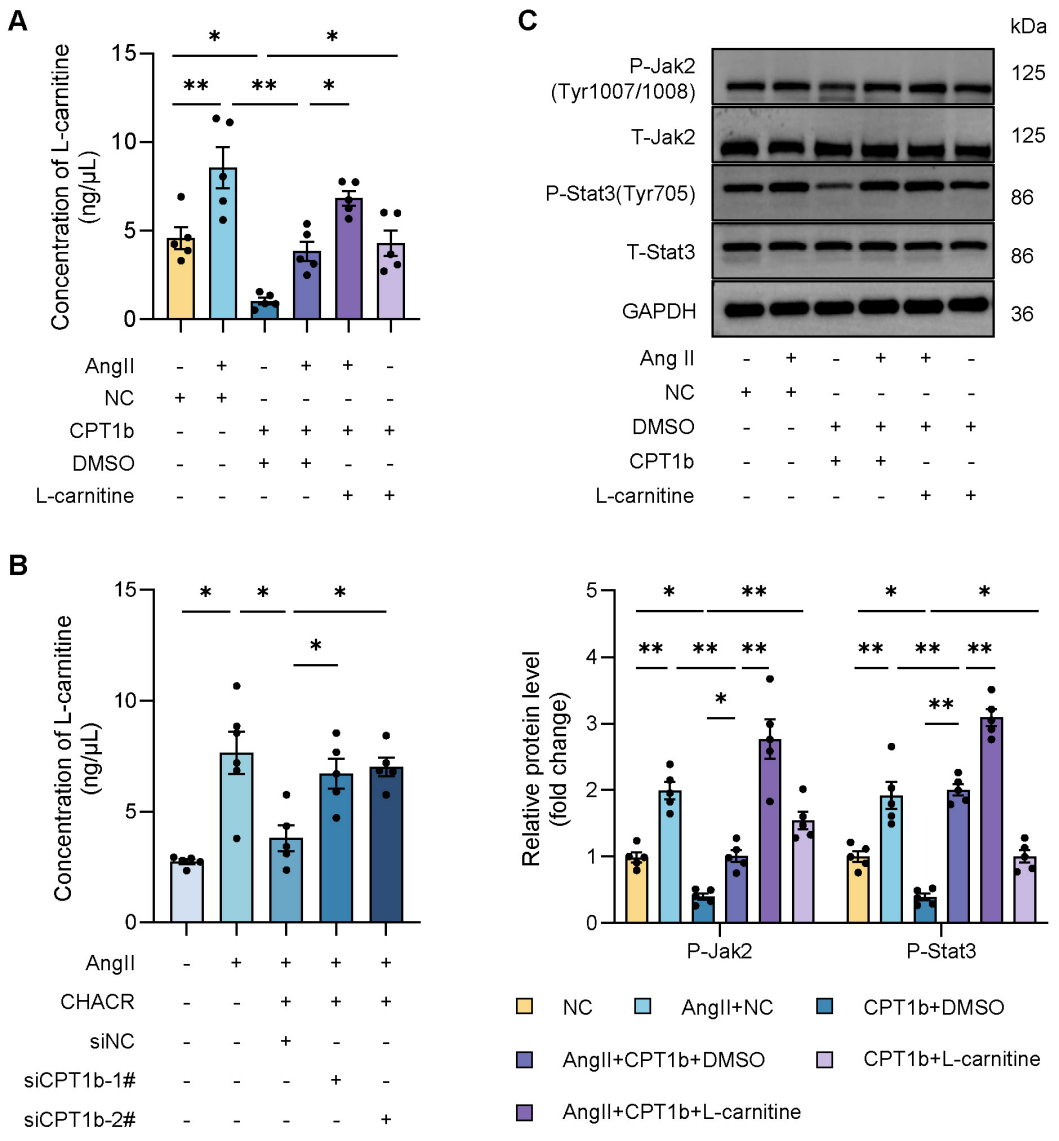
** The CPT1b inhibits the Jak2/Stat3 pathway by regulating L-carnitine level in cardiomyocytes.** (**A**) Quantification of L-carnitine in cardiomyocytes transfected with CPT1b with or without L-carnitine supplement (20 μg/mL) (n = 5). (**B**) Quantification of L-carnitine in cardiomyocytes transfected with transfected with CHACR or siCPT1b (n = 5). (**C**) Western blot results showing P-Jak2 (Tyr1007/1008) and P-Stat3 (Tyr705) expression in cardiomyocytes transfected with CPT1b with or without L-carnitine supplement (20 μg/mL) (n = 5). *p < 0.05, **p < 0.01.

Previous researches hinted that CPT1 was closely associated with L-carnitine level, which was involved in various signaling pathway including Stat3. Therefore, we sought to explore whether CPT1b regulated Jak2/Stat3 pathway by L-carnitine. It was found that L-carnitine level was significantly increased in Ang II-induced cardiomyocytes, which was decreased in CPT1b overexpressing hypertrophic cells, while L-carnitine supplement re-elevated the amount of L-carnitine (**Fig. 6A**), suggesting that CPT1b could suppress L-carnitine content and exogenous L-carnitine increased intracellular level. In addition, we also observed that CHACR downregulated L-carnitine by regulating CPT1b expression (**Fig. 6B**). To further confirm the role of L-carnitine in Jak2/Stat3 pathway, we detected the phosphorylation levels of Jak2 (Tyr1007/1008) and Stat3 (Tyr705) in cells supplying L-carnitine. We discovered that L-carnitine regulated by CPT1b expression could activate the Jak2/Stat3 signaling pathway (**Fig. 6C, S3**). Thus, we deemed that CPT1b might inhibit Jak2/Stat3 signaling pathway by decreasing L-carnitine level in cardiomyocytes.

### The CHACR attenuates myocardial hypertrophy by facilitating CPT1b expression in mice

**Figure 7:**
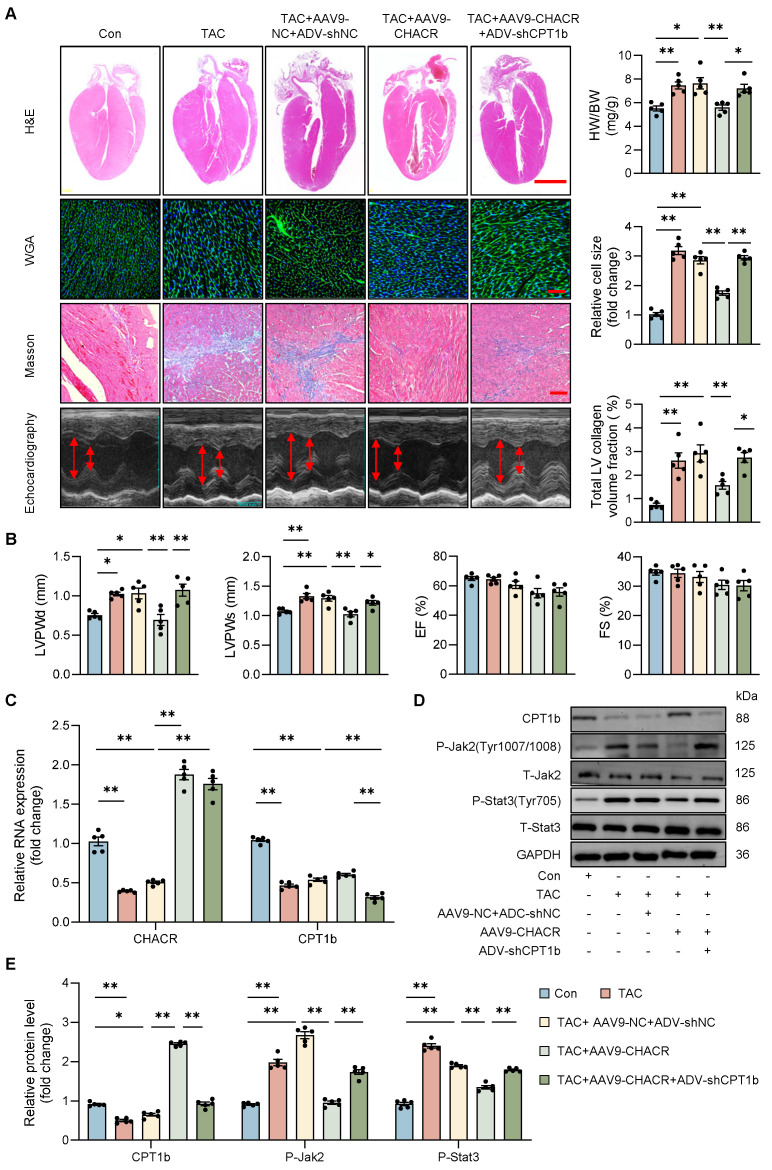
** The CHACR/CPT1b regulates cardiac hypertrophy *in vivo***. (**A**) Pathological changes in myocardial tissue evaluated using hematoxylin and eosin (H&E), wheat germ agglutinin (WGA), and Masson's staining. Heart weight/body weight ratio (HW/BW) and cardiomyocyte cross-section were calculated. Scale bar, 2 mm, 100 μm, and 100 μm, respectively (n = 5). **(B)** Left ventricular posterior wall thickness at end-diastole (LVPWd, mm), left ventricular posterior wall thickness at end-systole (LVPWs, mm), ejection fraction (EF, %), and fractional shortening (FS, %) were measured by echocardiography 4 weeks after TAC (n = 5). (**C**) qRT-PCR was used to detect the expression levels of CHACR and CPT1b in mouse hearts (n = 5). (**D**) Western blotting was used to measure CPT1b, P-Jak2 (Tyr1007/1008), and P-Stat3 (Tyr705) protein levels in mouse hearts. (**E**) Quantitative analysis of western blotting in (**D**) (n = 5). *p<0.05, **p<0.01.

*In vivo* studies were conducted to evaluate the roles of CHACR and CPT1b in pathological cardiac hypertrophy. Mice subjected to TAC showed notable cardiac hypertrophy and myocardial fibrosis, which were alleviated by overexpressing CHACR, as demonstrated by a decrease in heart-to-body weight ratio, relative cell area, ventricular wall thickness, and left ventricular collagen volume. In addition, the inhibition of CPT1b expression aggravated myocardial hypertrophy in TAC mice with overexpression of CHACR (**Fig. 7A, B**). Furthermore, CHACR inhibited the Jak2/Stat3 pathway by regulating CPT1b expression, which may be involved in the pathogenesis of cardiac hypertrophy (**Fig. 7C-E, S2**). In addition, CHACR or CPT1b overexpression alone in mice subjected TAC reduced cardiac hypertrophy compared to TAC+NC group (**Fig. S4**).

## Discussion

The present study demonstrated that the novel circRNA CHACR suppressed cardiac hypertrophy by facilitating the protein expression of CPT1b. CHACR, which was mainly expressed in the cytoplasm, bound directly to CPT1b and upregulated its expression, which consequently inhibited L-carnitine level to inactivate the Jak2/Stat3 pathway, thereby attenuating cardiac hypertrophy (**Graphical abstract**). The finding that CHACR/CPT1b inhibited the hypertrophic response represents a significant breakthrough in circRNA research on pathological cardiac remodeling.

Several studies have demonstrated the involvement of circRNAs in the pathogenesis of myocardial hypertrophy, via sponging miRNAs 23-25; however, an RNA pull-down assay in this study revealed that CHACR did not bind to AGO-2, as an indicator protein for circRNAs to function as sponges. Our findings suggested that CHACR inhibited cardiac hypertrophy in mice by binding to and regulating the expression of CPT1b protein. CircRNA-protein interactions can affect the expression and biogenesis of proteins 26,27. In this study, we found that CHACR interacted with CPT1b and protected it from proteasomal degradation by reducing its ubiquitination level in cardiomyocytes, thus alleviating cardiac hypertrophy.

CPT1b is a member of the CPT1 family that is mainly located in the outer mitochondrial membrane and is the pivotal rate-limiting enzyme of long-chain fatty acid synthesis and oxidation in myocardial mitochondria. As the main isoform of adult cardiomyocytes, CPT1b represents 98% of the total CPT1 cardiac activity 28,29. Importantly, the lack of CPT1b does not affect the normal heart but aggravates myocardial hypertrophy and cardiac dysfunction caused by pressure overload 22. Consistent with our findings that CPT1b overexpression attenuated cardiomyocyte hypertrophy induced by Ang II, it has been shown that the binding of enhancer of zeste homolog 2 to the promoter of CPT1b downregulated the expression of CPT1b, leading to cardiomyocyte hypertrophy aggravation 21. Many researchers have focused on the functions of CPT1b in cardiomyocytes, especially fatty acid oxidation, which is crucial in improving resistance to hypoxia and stimulating cardiomyocyte proliferation 20,22,29. It was found that CPT1b did promote fatty acid oxidation, in line with those researches. However, the molecular mechanism of CPT1b in cardiomyocytes remains unclear. Therefore, the present study sought to explore the regulatory mechanism of CPT1b in the pathogenesis of cardiomyocyte hypertrophy.

KEGG pathway analysis on RNA pull-down materials hinted us that JAK signaling pathway were involved. As is known, activation of Jak2/Stat3 signaling pathway would lead to the production of factors such as ANP/BNP, which are vital in cardiac hypertrophy 30,31. Stat3 is regarded as a crucial regulator during hypertrophic growth 32,33. The activity of Stat3 is strictly regulated in physiological processes, and its abnormal and persistent activation leads to pathological cardiac hypertrophy 34,35. On this foundation, we proved that CPT1b suppressed Jak2/Stat3 signaling pathway to aggravate cardiac hypertrophy. As one of carnitine palmitoyl-transferases, CPT1b can convert long-chain acyl CoA and carnitine in cytoplasm into acylcarnitine equivalents, which are transported to the mitochondrial inner membrane via carnitine acylcarnitine transferase. Also, CPT1b knockout led to an increase of carnitine content in cardiomyocytes 20. And, as the only biologically active carnitine, many studies have found that L-carnitine regulated various signaling pathway, such as promoting Stat3 activation 36, activating the AKT/FOXO3a pathway 37 and facilitating p38 MAPK/Nrf2 signaling in hearts 38,39. Therefore, we deemed that CPT1b might inhibit Jak2/Stat3 signaling pathway by reducing L-carnitine level in cardiomyocytes. Certainly, the specific mechanism of L-carnitine needs further exploration. Zhang *et al.* found that Coumermycin A1 induced JAK2 signaling activation and increases the expression of CPT1b in mouse adipocytes 40, however, we confirmed that CPT1b did regulate the phosphorylation levels of Jak2 and inhibiting Jak2/Stat3 signaling pathway made no effects on CPT1b expression in cardiomyocytes.

## Conclusions

CHACR attenuated cardiac hypertrophy by increasing the expression of CPT1b, which is involved in regulation of the Jak2/Stat3 pathway, demonstrating the participation of this circRNA-mediated protein mechanism in the pathogenesis of cardiac hypertrophy. CHACR may thus be a potential therapeutic target for pathological myocardial hypertrophy.

## Supplementary Material

Supplementary figures and tables.
