# Supplementary material for: Circular RNA CHACR is involved in the pathogenesis of cardiac hypertrophy
Source: Theranostics. 2025 Feb 26;15(8):3627–42. doi: 10.7150/thno.104695 (PMC11905130; doi:10.7150/thno.104695)
Supplement: Supplementary file 1 — Supplementary figures and tables. [file thnov15p3627s1.pdf]

# Supplementary materials

**Table S1. Primer sequences of genes for RT-qPCR**

| Primers | Forward | Reverse |
|---|---|---|
| CHACR | ACGGTGTCCTGAAACCTGAT | ACCATTGTCCCGAATCTCAA |
| ANP | CCAGCATGGGCTCCTTCTCCA | CCGGAAGCTGTTGCAGCCTAGT |
| β-MHC | CCGAGTCCCAGGTCAACAA | CTTCACGGGCACCCTTGGA |
| BNP | TAGCCAGTCTCCAGAGCAATTC | TTGGTCCTTCAAGAGCTGTCTC |
| CPT1b | CACCAGGCAGTAGCTTTCCAGTTC | AGGCGTTTCTTCCAGGAGTTGATTC |
| GAPDH | AAGGTCATCCCAGAGCTGAA | CTGCTTCACCACCTTCTTGA |
| Divergent | ACGGTGTCCTGAAACCTGAT | ACCATTGTCCCGAATCTCAA |
| Convergent | CGCCTTGAACCAGAGACTCT | CATGGGCTTCTTGACTGCTC |
| ACTB | AGTGTGACGTTGACATCCGT | TGCTAGGAGCCAGAGCAGTA |

**Table S2. Sequences of CHACR**

**> chr1: 53256629-53282092**

CTACTCAGGTGCAAGCTAGGATGAAACAGTTGCCTGCAGCAACAGTTCGCCTCCTGTCCAGTT
CTCAGACAATCACGTCAGTGGTCAGCGTTGTGAAAGAGCTCATTGAAAACTCCTTGGATGCCG
GAGCCACCAGCATTGAAGTTAAACTGGAGAACTATGGATTTGATAAAATTGAGATTCGGGACAA
TGGTGAGGGCATCAAGGCTGTAGATGTCCCTGTAATGGCAGTGAAGTACTACACCTCGAAGAT
CAGCAGTCATGAAGACCTTGGAAATCTGACAACTTATGGTTTTCGTGGTGAAGCCTTGGGGTC
AATATGTAATGTTGCGGAGGTGGTAGTTACAACAAGGACATCTGCTGATGACTTTAGCACTCAG
TATGTTTTAGATGGCAGTGGCCACATACTTTCTCAGAAGCCTTCACATCTTGGTCAAGGTACAA
CTGTAACTGCTCTAAAGTTGTTTAAGAATCTGCCTGTAAGAAAACAATTTTACTCAACAGCTAAA
AAGTGTAAAGATGAACTAAAAAATGTACAGGACCTTCTCATAAGCTACGGTGTCCTGAAACCTG
ATGTGAGGATTACTTTTGTACATAATAAG

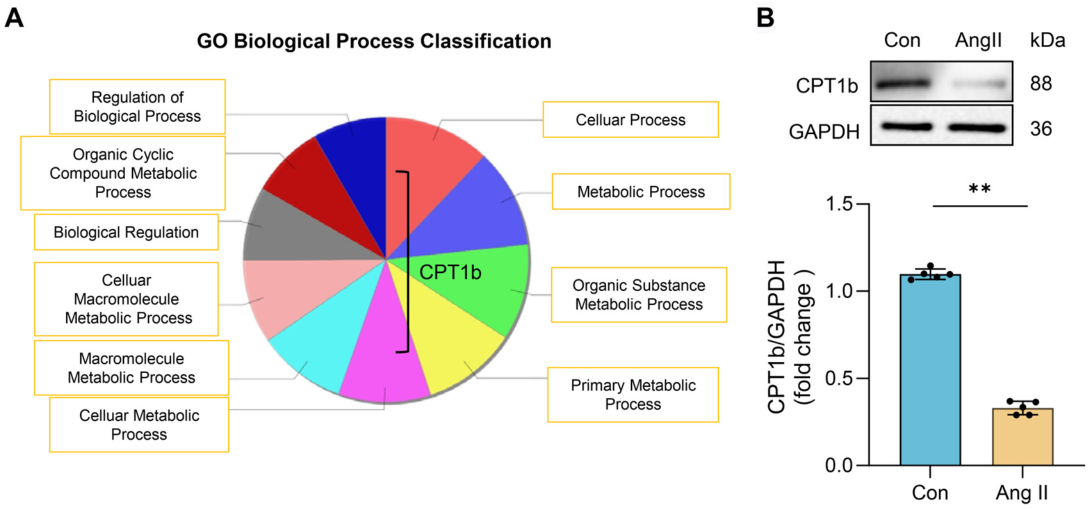


**Figure S1. CPT1b was involved in CHACR-regulated cardiomyocyte hypertrophy.** (**A**) Gene Ontology-based biological process classification of CPT1b. (**B**) Western blot results for CPT1b expression in cardiomyocytes with or without Ang II treatment (1 μg/mL) (n = 5). **$p < 0.01$.

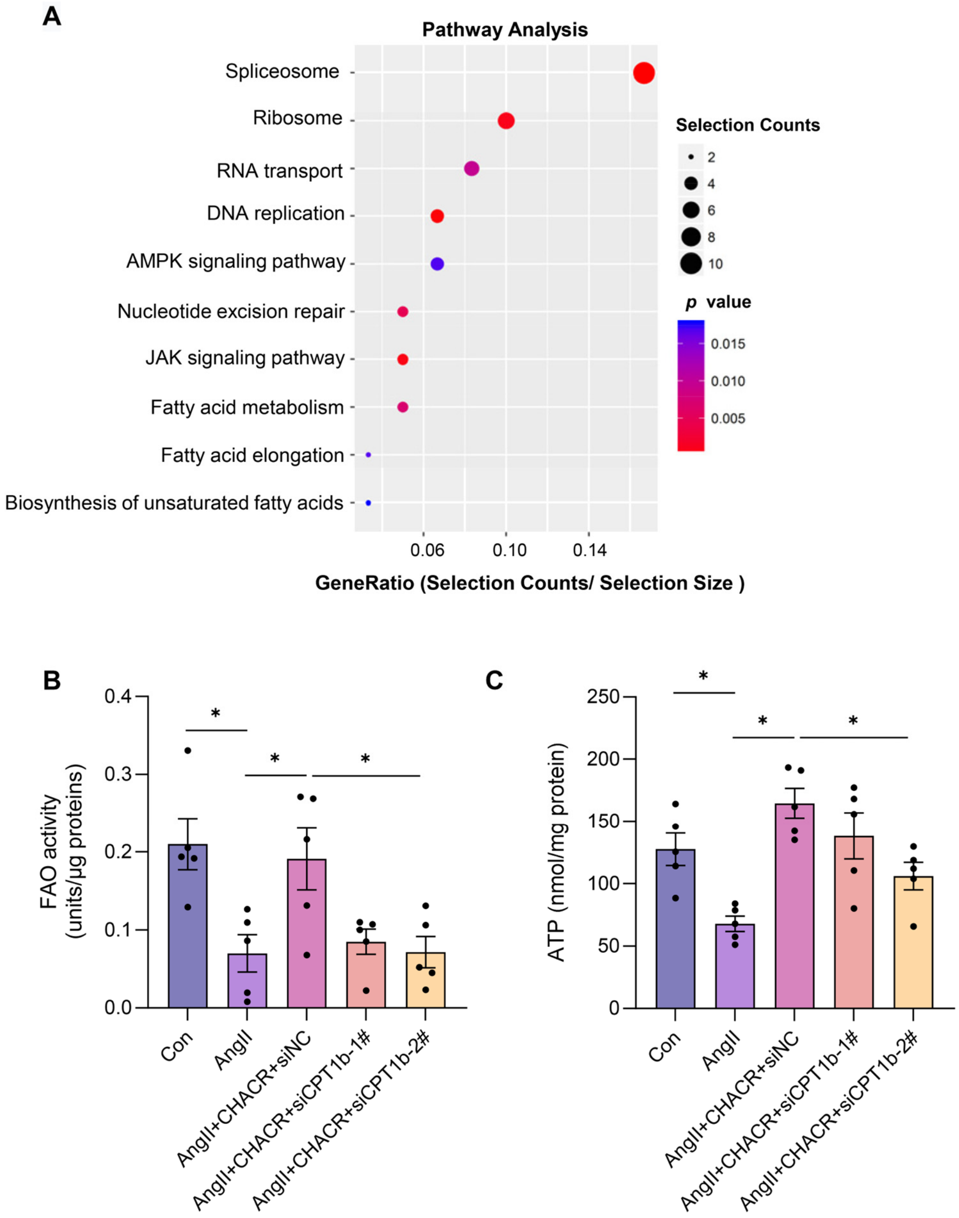


**Figure S2. CHACR promote fatty acid oxidation (FAO) and ATP production in cardiomyocytes.** (**A**) KEGG pathway analysis on the bound protein in the RNA pull-down materials. (**B**) The measurement of FAO activity in cardiomyocytes stimulated with Ang II (1 μg/mL) and transfected with CHACR and/or siCPT1b (n = 5). (**C**) The quantification of ATP in cardiomyocytes stimulated with Ang II (1 μg/mL) and transfected with CHACR and/or siCPT1b (n = 5). *$p < 0.05$.

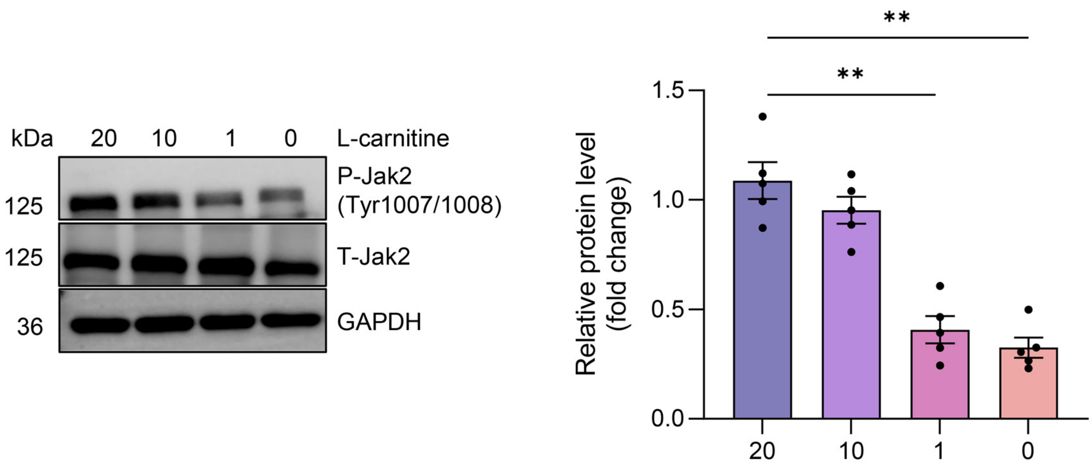


**Figure S3. L-carnitine treatment activated P-Jak2 (Tyr1007/1008) expression in cardiomyocytes.** Western blot results showing P-Jak2 (Tyr1007/1008) expression in cardiomyocytes with L-carnitine treatment in different concentration (DMSO, 1 μg/mL, 10 μg/mL, 20 μg/mL). **p < 0.01.

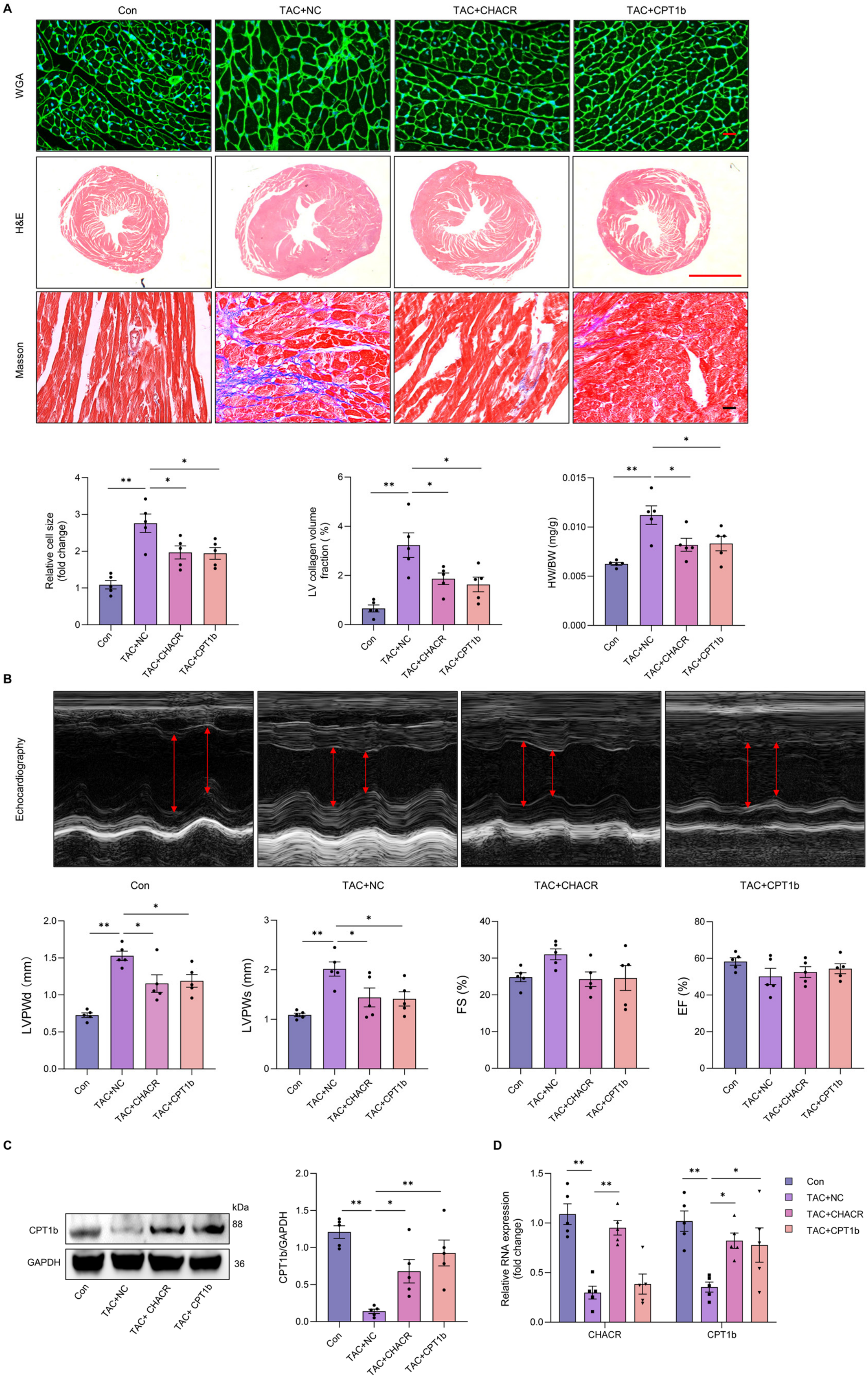
A
Con
TAC+NC
TAC+CHACR
TAC+CPT1b
WGA
H&E
Masson
Relative cell size (fold change)
LV collagen volume fraction ( %)
HW/BW (mg/g)
B
Echocardiography
LVPWd (mm)
LVPWs (mm)
FS (%)
EF (%)
C
CPT1b
GAPDH
kDa
88
36
TAC+ CHACR
TAC+ CPT1b
CPT1b/GAPDH
D
Relative RNA expression (fold change)
CHACR
CPT1b

**Figure S4. The CHACR or CPT1b overexpression alleviated cardiac hypertrophy *in vivo*.** (**A**) Pathological changes in myocardial tissue evaluated using wheat germ agglutinin (WGA), hematoxylin and eosin (H&E) and Masson's staining. Heart weight/body weight ratio (HW/BW), cardiomyocyte cross-section and collagen volume fraction were calculated. Scale bar, 100 μm, 2 mm, 100 μm, respectively (n = 5). (**B**) Left ventricular posterior wall thickness at end-diastole (LVPWd), left ventricular posterior wall thickness at end-systole (LVPWs), ejection fraction (EF%), and fractional shortening (FS%) were measured by echocardiography 4 weeks after TAC (n = 5). (**C**) Western blotting was used to measure CPT1b protein levels in mouse hearts. (**D**) qRT-PCR was used to detect the expression levels of CHACR and CPT1b in mouse hearts (n = 5). $^{*}p < 0.05$, $^{**}p < 0.01$.
